# Ambulatory heart rate range predicts mode-specific mortality and hospitalisation in chronic heart failure

**DOI:** 10.1136/heartjnl-2015-308428

**Published:** 2015-12-16

**Authors:** Richard M Cubbon, Naomi Ruff, David Groves, Antonio Eleuteri, Christine Denby, Lorraine Kearney, Noman Ali, Andrew M N Walker, Haqeel Jamil, John Gierula, Chris P Gale, Phillip D Batin, James Nolan, Ajay M Shah, Keith A A Fox, Robert J Sapsford, Klaus K Witte, Mark T Kearney

**Affiliations:** 1 Multidisciplinary Cardiovascular Research Centre, LIGHT Laboratories, The University of Leeds, Leeds, UK; 2 Medical Physics and Clinical Engineering Department, Royal Liverpool University Hospital, Liverpool, UK; 3 Physics Department, University of Liverpool, Liverpool, UK; 4 Cardiology Department, Pinderfields General Hospital, Mid Yorkshire Hospitals NHS Trust, Wakefield, UK; 5 University Hospital of North Staffordshire, Stoke-on-Trent, UK; 6 BHF Centre of Excellence, King's College London, London, UK; 7 BHF Centre for Cardiovascular Science, University of Edinburgh, Edinburgh, UK; 8 Cardiology Department, Leeds General Infirmary, Leeds Teaching Hospitals NHS Trust, Leeds, UK

## Abstract

**Objective:**

We aimed to define the prognostic value of the heart rate range during a 24 h period in patients with chronic heart failure (CHF).

**Methods:**

Prospective observational cohort study of 791 patients with CHF associated with left ventricular systolic dysfunction. Mode-specific mortality and hospitalisation were linked with ambulatory heart rate range (AHRR; calculated as maximum minus minimum heart rate using 24 h Holter monitor data, including paced and non-sinus complexes) in univariate and multivariate analyses. Findings were then corroborated in a validation cohort of 408 patients with CHF with preserved or reduced left ventricular ejection fraction.

**Results:**

After a mean 4.1 years of follow-up, increasing AHRR was associated with reduced risk of all-cause, sudden, non-cardiovascular and progressive heart failure death in univariate analyses. After accounting for characteristics that differed between groups above and below median AHRR using multivariate analysis, AHRR remained strongly associated with all-cause mortality (HR 0.991/bpm increase in AHRR (95% CI 0.999 to 0.982); p=0.046). AHRR was not associated with the risk of any non-elective hospitalisation, but was associated with heart-failure-related hospitalisation. AHRR was modestly associated with the SD of normal-to-normal beats (R^2^=0.2; p<0.001) and with peak exercise-test heart rate (R^2^=0.33; p<0.001). Analysis of the validation cohort revealed AHRR to be associated with all-cause and mode-specific death as described in the derivation cohort.

**Conclusions:**

AHRR is a novel and readily available prognosticator in patients with CHF, which may reflect autonomic tone and exercise capacity.

## Introduction

Resting heart rate offers important prognostic information in patients with chronic heart failure (CHF), and its reduction is an important target during the titration of mortality-reducing pharmacotherapy.^1^ ^2^ It is also recognised that beat-to-beat variations in heart rate offer additional prognostic information, probably by reflecting autonomic dysfunction that contributes to the progression of CHF.^3^ ^4^ However, assessment of heart rate variability (HRV) using ambulatory electrocardiography is complex and limited to patients with sustained periods of normal sinus rhythm,^5^ potentially excluding more than half of the CHF population. We therefore set out to define whether variation in ambulatory heart rate during a 24 h period represents a simpler and more generalisable prognostic marker.

## Methods

We conducted a prospective cohort study aiming to define prognostic markers in patients with CHF, associated with left ventricular systolic dysfunction (LVSD), receiving contemporary evidence-based therapies. Between June 2006 and December 2011, all patients attending specialist cardiology clinics in four UK hospitals were approached to participate.^6^ In total, 1091 recruited patients provided written informed consent, and the Leeds West Research Ethics Committee gave ethical approval; the investigation conforms with the principles outlined in the *Declaration of Helsinki*. All 791 patients with available ambulatory ECG data were included in this analysis. Inclusion in the study required the presence of stable signs and symptoms of heart failure for at least 3 months, and left ventricular ejection fraction ≤45% on transthoracic echocardiography; recruiting clinics reviewed adult patients only (age ≥18 years).

As described previously,^7^ details of medical history were collected at recruitment, and symptomatic status defined using the New York Heart Association (NYHA) classification. Venous blood was collected for measurement of electrolyte concentrations, assessment of renal function and haematological parameters; these were performed in the local hospital chemical pathology laboratories. Estimated glomerular filtration rate was calculated using the Modification of Diet in Renal Disease method.^8^ Two-dimensional echocardiography was performed according to British Society of Echocardiography recommendations.^7^ Resting heart rate was measured using 12-lead ECGs. Use of diuretic therapy, ACE inhibitors (ACEi), angiotensin receptor blockers and β-blockers were collected at study recruitment. The prescribed daily doses of β-blockers and diuretics were expressed relative to the maximal licensed dose of bisoprolol and furosemide, respectively.^7^ Receipt of cardiac resynchronisation therapy or implantable cardioverter-defibrillator was defined 6 months after recruitment to account for device implantation shortly after referral to the service. ‘Any device therapy’ refers to patients with either a pacemaker or defibrillator. ‘Any atrial fibrillation or flutter’ was defined as paroxysmal, persistent or permanent arrhythmia on medical history, 12-lead or Holter electrocardiograph. Peak oxygen uptake was measured as described,^9^ from the last 30 s of a symptom-limited incremental peak exercise test on a treadmill or stationary cycle, using breath-by-breath analysis (Medgraphics, Minnesota, USA).

### Ambulatory heart rate range and SD of normal-to-normal beats analyses

Twenty-four hour ambulatory three-lead ECGs (Lifecard CF, Spacelabs Healthcare, Washington, USA) were obtained during normal, unrestricted, out-of-hospital activity.^7^ Recordings were analysed with Delmar Reynolds Pathfinder or Spacelabs Sentinel Systems by independent technical staff blinded to patient characteristics. Each 24 h ECG recording was manually edited to exclude incorrectly identified R waves and include unidentified R waves as determined at the automatic processing stage. Ambulatory heart rate range (AHRR) was calculated as maximum minus minimum heart rate during the period of analysis; automated mean heart rates for each minute of the day (including all paced and non-sinus complexes) were used to derive this data. For SD of normal-to-normal beats (SDNN) analyses, the standard Delmar Reynolds/Spacelabs RR interval exclusion criteria were then applied to the manually edited records. Specifically, RR intervals were excluded if: RR >2.0 s; RR>3 SDs of the local 20 min interval; RR intervals <300 ms; RR>120% of previous RR; RR <80% of previous RR. SDNN was then determined using the proprietary Pathfinder software. Ambulatory ECG data collected during a follow-up clinic visit approximately 1 year later (available for 328 patients) were used to define changes in ambulatory heart rate parameters.

### Mortality and hospitalisation data

All patients were registered with the UK Office of Population Censuses and Surveys, which provided details of death. Classification criteria for the mode of death were defined before the study commenced, based upon previous publications.^10^ At least two senior physicians reviewed each death certificate and gathered data as required from autopsy reports, hospital notes and primary care records. Mode of death was classified as: (1) sudden cardiac, if it occurred within 1 h of a change in symptoms or during sleep or while the patient was unobserved (defibrillator therapies were not included as a proxy); (2) progressive HF, if death occurred after a documented period of symptomatic or haemodynamic deterioration; (3) other cardiovascular death, if not occurring suddenly or in association with progression of HF (eg, cerebrovascular accident); (4) non-cardiovascular death; and (5) unclassifiable, where insufficient information was available to reach a firm conclusion. Heart-failure-related hospitalisation was assessed as described,^7^ using institutional clinical event databases detailing all admissions in recruiting centres. Details of all non-elective hospitalisations were also collected. Events were assessed during the first year of recruitment and analysed as a binary outcome.

### Validation cohort

In order to assess the validity of our observations, analyses were repeated in a second cohort of patients from the previously published UK-HEART study.^4^ Briefly, 553 patients with CHF associated with preserved or reduced ejection fraction were recruited between December 1993 and April 1995, with the aim to assess the prognostic value of HRV analysis; of these 408 had 24 h ECG data suitable for analysis. Patients with atrial arrhythmias were excluded from this study due to their preclusion of HRV analyses. No patients included in this analysis had implantable cardiac devices.

### Statistics

All analyses were conducted with SPSS V.21 (IBM, Armonk, New York, USA). Continuous data are displayed as mean (SE of mean), and categorical data are displayed as percentage (number); normality of distribution was confirmed on skewness testing. Continuous data are compared with unpaired Student's t tests, or analysis of variance (ANOVA) as appropriate, and categorical data with χ^2^ tests. Cox proportional hazards regression analysis was used in univariate and multivariate mortality analyses (using all variables listed in table 3, without stepwise elimination), and univariate hospitalisation analyses. Assessment of partial residuals was used to confirm no deviation from the assumption of proportional hazards. HRs are presented with 95% CIs. The relationship between AHRR and SDNN or VO_2_max was defined with Pearson's correlation test. Statistical significance was defined as p<0.05.

## Results

Descriptive data for the 791 patients with available ambulatory ECG data, divided into quartiles of AHRR (median 47; IQR 36–59), are provided in table 1. Lower AHRR was associated with older age, lower resting heart rate, broader QRS interval, lower haemoglobin, worse renal function, more ischaemic heart disease, more diabetes, worse functional capacity, more device therapy and less atrial dysrhythmia. Differences in AHRR between these groups appeared to be predominantly due to differences in maximum heart rate.

**Table 1:** Cohort characteristics

Variable	AHRR <36 (n=181)	AHRR 36–46 (n=202)	AHRR 47–58 (n=197)	AHRR ≥59 (n=211)	p Value (across all groups)
Age (years)	71.1 (0.8)	69.1 (0.8)	67.8 (0.9)	63.9 (0.9)	<0.001
Resting heart rate (bpm)	70 (2)	71 (1)	73 (1)	80 (1)	<0.001
Systolic blood pressure (mm Hg)	121 (2)	121 (2)	121 (2)	123 (2)	0.71
QRS interval (ms)	126 (2)	125 (2)	122 (2)	116 (2)	0.01
Haemoglobin (g/dL)	129 (1)	136 (1)	136 (1)	142 (1)	<0.001
Sodium (mmol/L)	139 (1)	139 (1)	140 (1)	140 (1)	0.12
eGFR (mL/Kg/1.73 m^2^)	49 (1)	54 (1)	56 (1)	61 (1)	<0.001
LV ejection fraction (%)	33 (1)	32 (1)	32 (1)	31 (1)	0.58
Minimum 24 h heart rate (bpm)	59 (1)	58 (1)	56 (1)	57 (1)	0.37
Maximum 24 h heart rate (bpm)	86 (1)	98 (1)	108 (1)	133 (1)	<0.001
Ambulatory heart rate range (bpm)	28 (1)	41 (1)	52 (1)	76 (1)	<0.001
Male sex (% (n))	73.5 (133)	73.8 (149)	74.1 (146)	73.5 (155)	0.99
Ischaemic aetiology (% (n))	79 (143)	69.3 (140)	66 (130)	42.2 (89)	<0.001
NYHA class					0.12
I	14.4 (26)	17.9 (36)	25.4 (50)	24.3 (51)	
II	48.1 (87)	46.3 (93)	39.6 (78)	48.1 (101)	
III	35.4 (64)	33.3 (67)	33.5 (66)	26.7 (56)	
IV	2.2 (4)	2.5 (5)	1.5 (3)	1 (2)	
Any device therapy (% (n))	36.5 (66)	32.2 (65)	24.9 (49)	20.4 (43)	0.002
Cardiac resynchronisation (% (n))	28.7 (52)	28.7 (58)	23.4 (46)	19 (40)	0.06
Implantable defibrillator (% (n))	18.2 (33)	13.4 (27)	8.6 (17)	9 (19)	0.01
Any atrial fibrillation or flutter (% (n))	22 (39)	23.9 (48)	30.4 (58)	48.8 (100)	<0.001
Non-sustained VT (% (n))	36.9 (65)	35.8 (72)	37.6 (71)	47.1 (96)	0.08
Diabetes (% (n))	40.9 (74)	28.7 (58)	20.8 (41)	14.7 (31)	<0.001
β-blocker use (% (n))	82.3 (149)	81 (162)	77.2 (152)	76.6 (160)	0.42
β-blocker dose (mg bisoprolol/day)	4.0 (0.3)	3.4 (0.2)	3.6 (0.2)	3.1 (0.2)	0.088
ACEi/ARB use (% (n))	86.7 (157)	87 (174)	91.4 (180)	87.6 (183)	0.45
MRA use (% (n))	45.9 (83)	43.5 (87)	41.1 (81)	32.1 (67)	0.03
Furosemide dose (mg/day)	66 (4)	56 (4)	52 (4)	40 (3)	<0.001

AHRR, ambulatory heart rate range; ACEi, ACE inhibitors; ARB, angiotensin receptor blockers; eGFR, estimated glomerular filtration rate; LV, left ventricular; MRA, mineralocorticoid receptor antagonist; NYHA, New York Heart Association; VT, ventricular tachycardia.

### Mortality and hospitalisation analyses

**Figure 1:**
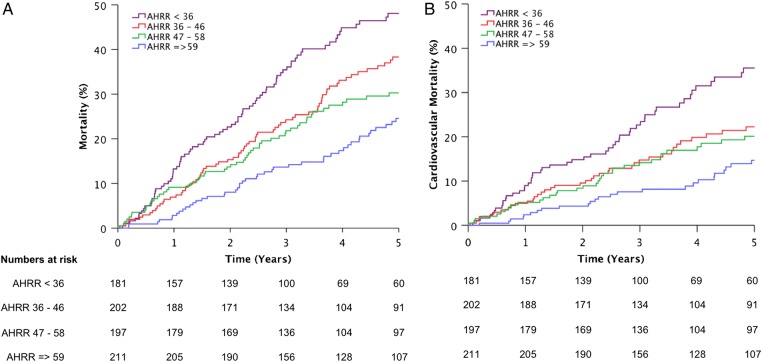
All-cause mortality and AHRR. Kaplan–Meier curves illustrating all-cause (A) and cardiovascular (B) mortality according to quartiles of AHRR (p<0.001 by log-rank analysis). AHRR, ambulatory heart rate range.

After a mean follow-up period of 4.1 years (SE 0.07 years), a total of 268 deaths occurred (95 progressive heart failure, 43 sudden, 104 non-cardiovascular, 17 other cardiovascular, 9 not classifiable). As illustrated in figure 1, all-cause and cardiovascular mortality increased across quartiles of declining AHRR. There was no interaction between the presence of atrial arrhythmia and AHRR in predicting all-cause mortality (HR for no atrial arrhythmia group 0.971 (95% CI 0.961 to 0.982); HR for atrial arrhythmia group 0.984 (0.975 to 0.994)). There was no interaction between the use of device therapy and AHRR in predicting all-cause mortality (HR for no device group 0.981 (95% CI 0.972 to 0.989); HR for device group 0.981 (0.969 to 0.994)). Importantly, AHRR was also associated with risk of progressive heart failure death, sudden death and non-cardiovascular death in univariate analyses (table 2). After accounting for characteristics that differed between AHRR quartiles (with p≤0.002 to account for multiple tests) and bisoprolol dose (as a potential confounder) using multivariate analysis, AHRR remained associated with all-cause mortality (0.9% reduction in mortality over the whole study period per beat/minute increase in AHRR (95% CI 0% to 1.8%); p=0.046; table 3).

**Table 2:** Univariate mortality analyses

		95% CI of HR		
Mode of death	HR	Low	High	p Value
All cause	0.981	0.974	0.988	<0.001
Progressive heart failure	0.981	0.969	0.993	0.002
Sudden	0.972	0.954	0.990	0.003
Non-cardiovascular	0.981	0.97	0.993	0.001

HRs associated with a 1 bpm increase in AHRR

AHRR, ambulatory heart rate range.

**Table 3:** Multivariate analysis of all-cause mortality

		95% CI of HR			
Variable	HR	Low	High	p Value	Wald
Age (per year)	1.048	1.032	1.065	<0.001	34.2
Diabetes	1.75	1.31	2.35	<0.001	14.2
Haemoglobin (per 10 g/L)	0.87	0.8	0.95	0.002	9.5
Resting heart rate (per bpm)	1.011	1.004	1.019	0.004	8.4
Ischaemic cardiomyopathy	1.57	1.11	2.23	0.011	6.5
AHRR (per bpm)	0.991	0.982	0.999	0.046	4
Any atrial fibrillation or flutter	1.34	0.99	1.82	0.06	3.5
Bisoprolol daily dose (per mg)	0.97	0.92	1.01	0.12	2.4
Any device therapy	1.11	0.83	1.49	0.48	0.5
eGFR (per mL/Kg/1.73 m^2^)	1.003	0.994	1.013	0.49	0.5

AHRR, ambulatory heart rate range; eGFR, estimated glomerular filtration rate.

During the first year of follow-up, 180 patients underwent an unplanned hospitalisation, with 46 attributable to heart failure. AHRR was not associated with the risk of any non-elective hospitalisation (HR 0.993; 95% CI 0.985 to 1.001; p=0.079), but was associated with the risk of heart-failure-related hospitalisation (HR 0.977; 95% CI 0.96 to 0.994; p=0.009).

AHRR declined by 7.6% (49.8 (0.1) vs 46 (0.9) bpm; p<0.001) between recruitment and follow-up (after 354 (7) days) in the subset of 328 patients with repeat ambulatory ECG data. Notably, β-blocker titration in this period was not associated with either changes in AHRR or absolute AHRR at follow-up (table 4). Follow-up AHRR remained associated with all-cause mortality within this subgroup (HR 0.98; 95% CI 0.968 to 0.992; p=0.002).

**Table 4:** Changes in ambulatory heart rate range (AHRR) according to β-blocker titration

	β-blocker dose reduced (n=30)	β-blocker unchanged (n=119)	β-blocker increased (n=170)	p Value
Change in bisoprolol dose (mg/day)	−3.8 (0.5)	0 (0)	4.1 (0.2)	<0.001
Bisoprolol dose at follow-up (mg/day)	0.9 (0.3)	4.5 (0.3)	6.4 (0.2)	<0.001
Change in AHRR (bpm)	−2.8 (3.3)	−0.7 (1.5)	−5.1 (1.5)	0.13
AHRR at follow-up (bpm)	42.5 (2.5)	45.9 (1.5)	46.5 (0.9)	0.5

### Association of AHRR with functional capacity

**Figure 2:**
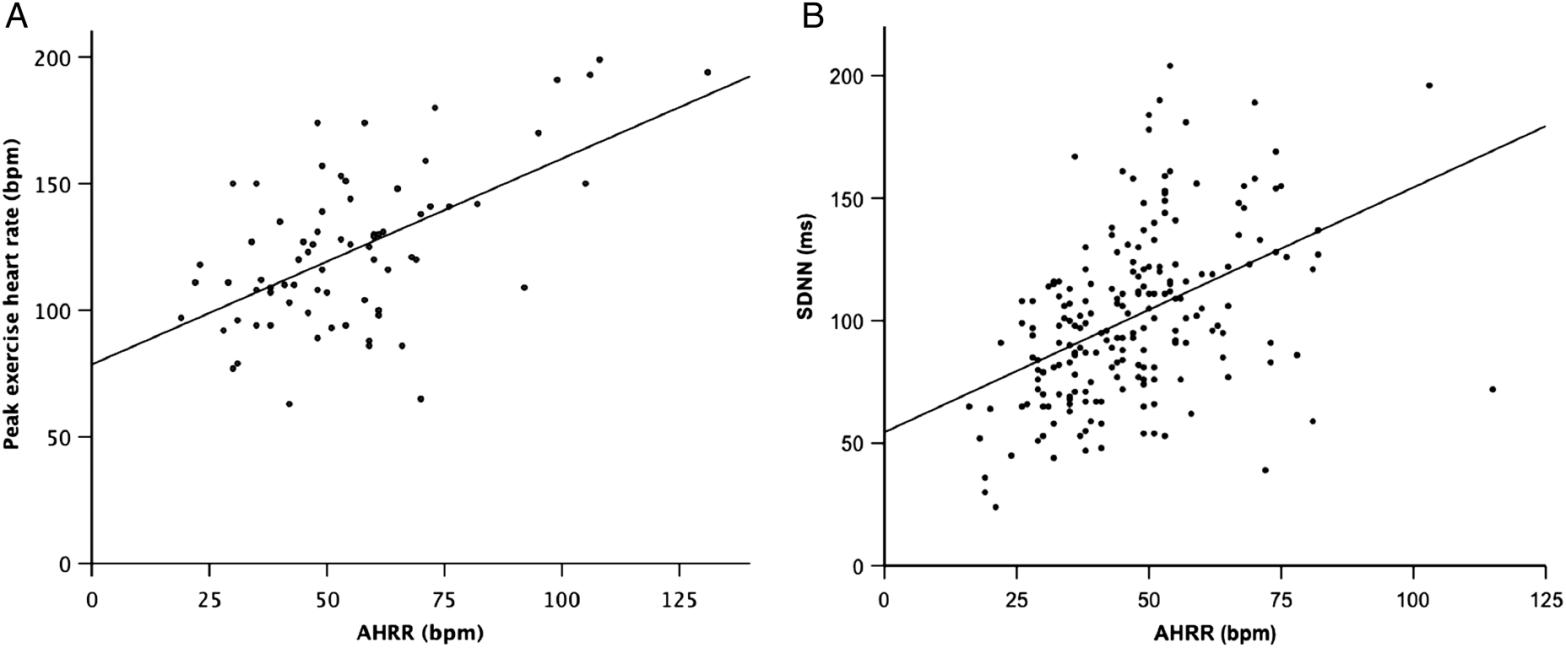
Correlative analyses. Scatter plots illustrating correlation between AHRR and: (A) maximal cardiopulmonary exercise test heart rate: R^2^ 0.33 (p<0.001); (B) SD of normal-to-normal beats: R^2^ 0.2 (p<0.001). AHRR, ambulatory heart rate range; SDNN, SD of normal-to-normal beats.

To explore what AHRR might tell us about patients with CHF, exploratory association analyses were performed. AHRR falls progressively as NYHA functional impairment classification rises (p=0.001 by ANOVA). Within the 98 patients in our AHRR cohort referred for cardiopulmonary exercise testing, there was very weak correlation between AHRR and VO_2_max (R^2^=0.06 (95% CI 0.001 to 0.20); p=0.015). However, differences in peak heart rate during cardiopulmonary exercise testing accounted for one-third of variation in AHRR (figure 2A; R^2^=0.33 (95% CI 0.1 to 0.55); p<0.001).

### Association of AHRR with HRV

Given the influence of the autonomic nervous system upon heart rate variation, we next explored the correlation between AHRR and SDNN. Data available for a subgroup of 199 patients collected as part of a separate substudy were included in this analysis; by definition, these patients were selected by virtue of being in sinus rhythm for sustained periods of monitoring. AHRR modestly correlated with SDNN (figure 2B; R^2^=0.2 (0.08 to 0.34); p<0.001). Interestingly, when both AHRR and SDNN were included in a multivariate analysis using these 199 highly selected patients, only SDNN was associated with risk of all-cause mortality (HRs (95% CI) SDNN 0.99 (0.981 to 0.998); AHRR 0.994 (0.976 to 1.012)).

### Validation cohort analyses

To assess the validity and generalisability of our observations, we conducted analyses in a cohort of patients with CHF associated with reduced or preserved left ventricular ejection fraction recruited in the late 1990s.^4^ The UK-HEART study aimed to define the prognostic role of HRV in CHF, and so only recruited patients without sustained atrial arrhythmia or other factors associated with autonomic dysfunction (diabetes, end-stage renal failure). Of the 553 patients recruited, 408 had 24 h ECG data of suitable quality to assess HRV, and so were included in this analysis. Mean age was 62.1 years (SE of mean 0.5), 74.5% were male, 77.9% had ischaemic aetiology and reduced left ventricular ejection fraction (below 50%) was noted in 64.8%. While 83.1% of patients received ACE inhibitor therapy, only 8.1% received β-blockers, in keeping with the study era. No patients had implantable cardiac devices. Hence, this cohort statistically differs (p<0.05) with our principal study cohort in terms of age, β-blocker use, ejection fraction, comorbidity and arrhythmia burden, allowing us to assess whether the prognostic value of AHRR transfers to more diverse CHF populations.

**Figure 3:**
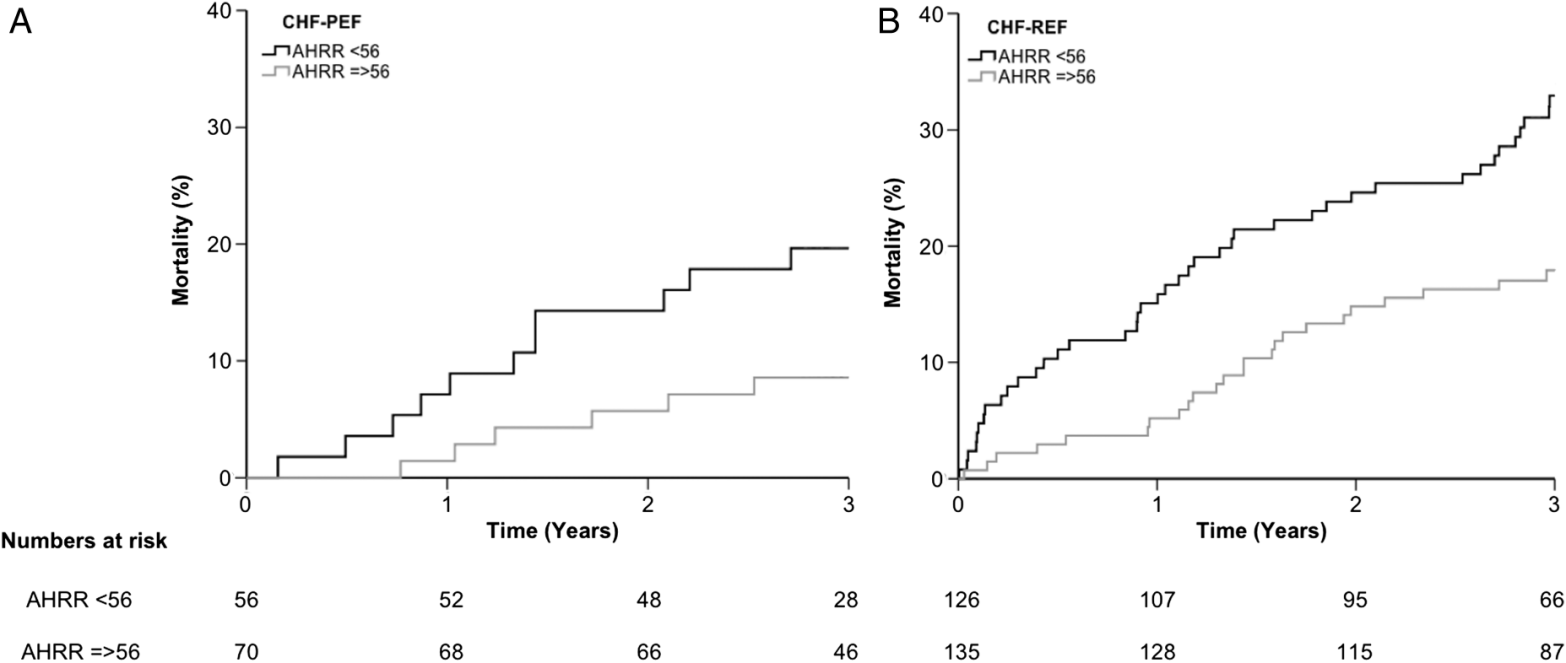
All-cause mortality in validation cohort. Kaplan–Meier curves illustrating all-cause mortality above or below median AHRR in patients with: (A) CHF-PEF; (B) CHF-REF (both p<0.05 by log-rank analysis). AHRR, ambulatory heart rate range; CHF-PEF, chronic heart failure with preserved ejection fraction; CHF-REF, chronic heart failure with reduced ejection fraction.

After a mean 3 years’ follow-up, 144 patients had died (59 progressive heart-failure-associated deaths, and 49 sudden deaths). In univariate analyses, AHRR remained associated with the risk of all-cause death (HR 0.973 (95% CI 0.962 to 0.984); p<0.001), progressive heart failure death (0.956 (0.939–0.974); p<0.001) and sudden death (0.981 (0.963 to 0.999); p=0.037). AHRR was associated with all-cause mortality in subgroups with reduced (n=261) or preserved (n=126) LV ejection fraction (figure 3). In a multivariate analysis identical to that presented in table 3 (other than without atrial arrhythmia, any device therapy or diabetes, since these were not present), AHRR remained strongly associated with the risk of all-cause death (0.968 (0.956 to 0.981); p<0.001). AHRR fell progressively with increasing NYHA class (p<0.001 by ANOVA), and correlated significantly with SDNN (R^2^=0.28 (0.19 to 0.36); p<0.001).

## Discussion

In this manuscript, we provide the first ever assessment of the prognostic value of the daily heart rate range in patients with CHF. To summarise, AHRR is associated with all-cause and mode-specific death, along with heart-failure-related hospitalisation; even after accounting for potentially confounding prognostic factors, AHRR remains associated with all-cause mortality. Notably, the relationship between AHRR and mortality persisted when assessed in an independently recruited cohort with CHF, within subgroups with either reduced or preserved left ventricular ejection fraction. The factors underpinning the association between AHRR and CHF outcome remain unclear, although our exploratory analyses indicate associations between AHRR and both functional capacity and autonomic dysfunction.

### Heart rate and prognosis in CHF

It is well established that elevated resting heart rate represents an important adverse prognostic factor, and also a therapeutic target, in patients with CHF associated with LVSD.^1^ ^2^ Moreover, a number of studies have suggested that the magnitude of heart rate reduction in response to β-blocker therapy is more important than the achieved dose of these agents in predicting outcome.^11^ ^12^ Importantly, researchers have long recognised the potentially useful additional prognostic data that can be derived from the dynamic properties of the heart rate, including, for example: chronotropic response to exercise,^13^ ^14^ heart rate recovery after exercise^15^ ^16^ and HRV.^3^ ^4^

SDNN, a marker of beat-to-beat variation in cycle length, is perhaps the best established measure of HRV in clinical research, and is recognised as a powerful prognosticator. However, it is also limited by the requirement of prolonged high-quality ECGs during uninterrupted sinus rhythm, essentially excluding more than half of a typical CHF population.^5^ Moreover, its derivation and interpretation is relatively complex, meaning that it is generally not applied in routine clinical practice. It is therefore interesting that AHRR is simple to derive in all patients within an unselected CHF cohort, and yet robustly predicts adverse outcome, possibly by reflecting some of the autonomic dysfunction we infer from SDNN. We feel that this simplicity may be critical in translating the use of AHRR as part of our prognostic repertoire in clinical practice, and allowing rapid assessment of its potential use in other settings, for example in implanted device monitoring.

### What does AHRR tell us?

While the data we present clearly suggest AHRR can assist in the prediction of mode-specific mortality and hospitalisation, we cannot offer an assessment of whether it acts as a marker or a mediator of adverse prognosis. However, our exploratory analyses suggest that AHRR is associated with important biological phenomena, which may themselves influence CHF outcome. First, we have demonstrated that as functional capacity and peak exercise heart rate decline, so too does AHRR. However, only a relatively small proportion of the variation in AHRR could be explained by peak exercise heart rate. Moreover, it remains unclear whether the link between AHRR and NYHA class is simply a reflection of physical inactivity, or whether this tells us something more about the disease process per se. A second biological link was between AHRR and autonomic tone, as assessed using SDNN—lower SDNN (an adverse prognostic feature) was noted as AHRR decreased. While it is interesting that a multivariate analysis including SDNN and AHRR suggested that only SDNN was associated with the risk of all-cause mortality, this analysis is limited by selection bias entailed in acquiring SDNN data. However, these analyses do add further support to the suggestion that autonomic tone is associated with AHRR, and notably diabetes is substantially more common in people with low AHRR. Interestingly, recently published data suggest that regular physical activity is also associated with increased HRV in older adults without CHF, potentially linking the phenomena we noted to be associated with AHRR.^17^

### Study limitations

While our observations are novel and have the potential to be readily adopted in routine clinical practice, they also have limitations. Our study population is relatively small, although it is important to note that this allowed us to phenotype our patients in more detail than is generally possible in large clinical trials (eg, SDNN analysis). It will therefore be important to further validate our findings in larger and more diverse CHF populations, although the recapitulation of our observations in a second CHF cohort adds significant strength to our findings. The observational nature of the study also prevents us from drawing any firm conclusions regarding the biological phenomena controlling AHRR, which is important in understanding its link to outcomes. Moreover, we are likely not to have accounted for all confounding factors in our multivariate analyses, but hope that these at least demonstrate that AHRR adds value to other readily available clinical data. In particular, we are unable to comment on NT-proBNP in our population or the value of AHRR as part of an established prognostic model. Finally, it will also be important to understand whether properties of the daily heart rate profile beyond its range offer further value.

### Remaining questions

As discussed earlier in this section, our data raise many other questions regarding the value of measuring AHRR during the assessment and management of patients with CHF. First, it will be important to assess the incremental value of AHRR beyond established prognostic scores (eg, the Seattle Heart Failure Model^18^). However, many factors included in established prognostic scores are already accounted for in our multivariate analysis, suggesting that AHRR may offer additional prognostic value. It will also be interesting to define how short-term and long-term changes in AHRR are associated with CHF events; this may suggest a role for AHRR monitoring by implantable cardiac devices, akin to previous data suggesting a role for SDNN monitoring.^19^ Finally, it will be interesting to explore whether AHRR during sleep offers prognostic value, and whether physical activity monitoring offers complementary data.

## Conclusions

AHRR is a simple to derive index of HRV during a 24 h period, which is associated with both indices of exercise physiology and autonomic function, and offers important prognostic information, independent of other established prognostic factors.

**Key messages**

**What is already known on this subject?**

- Resting heart rate and indices of heart rate variability are powerful predictors of adverse outcome in patients with chronic heart failure. However, most indices of heart rate variability are complex to derive and exclude patients without sustained periods of sinus rhythm.

**What might this study add?**

- The heart rate range during 24 h of ambulatory electrocardiography is associated with mode-specific mortality and hospitalisation in patients with heart failure, irrespective of left ventricular function or cardiac rhythm. After accounting for potential confounding factors, each one beat increase in heart rate range is associated with a 0.9% relative reduction in all-cause mortality.

**How might this impact on clinical practice?**

- While traditional heart rate variability indices offer valuable prognostic data, they are rarely applied in clinical practice due to complex analytical techniques and exclusion of patients with atrial arrhythmias and paced rhythms. Ambulatory heart rate range may circumvent these issues and offer other novel opportunities in the monitoring of patients with implantable cardiac devices.
